# Π-GISANS: probing lateral structures with a fan shaped beam

**DOI:** 10.1038/s41598-021-97112-x

**Published:** 2021-09-07

**Authors:** Alexei Vorobiev, Nicolò Paracini, Marité Cárdenas, Max Wolff

**Affiliations:** 1 Department for Physics and Astronomy, Uppsala University, Lägerhyddsvägen 1, 75120 Uppsala, Sweden; 2 Institute Laue-Langevin, 71 rue des martyrs, 38042 Grenoble, France; 3 Department of Biomedical Science and Biofilm Research Center for Biointerfaces, Malmö University, 20506 Malmö, Sweden

**Keywords:** Materials science, Condensed-matter physics, Soft materials, Techniques and instrumentation

## Abstract

We have performed grazing incidence neutron small angle scattering using a fan shaped incident beam focused along one dimension. This allows significantly reduced counting times for measurements of lateral correlations parallel to an interface or in a thin film where limited depth resolution is required. We resolve the structure factor of iron inclusions in aluminium oxide and show that the ordering of silica particles deposited on a silicon substrate depends on their size. We report hexagonal packing for 50 nm but not for 200 nm silica spheres deposited by a modified Langmuir-Schaefer method on a silicon substrate. For the 200 nm particles we extract the particles shape from the form factor. Moreover, we report dense packing of the particles spread on a free water surface. We name this method π-GISANS to highlight that it differs from GISANS as it gives lateral information while averaging the in-depth structure.

## Introduction

The understanding of interfacial properties of materials as well as interfacial processes in terms of structural, morphological and compositional changes is of crucial importance in many scientific fields that include chemistry, physics, biology and engineering. Overall, surface science has advanced enormously over the last decades, which is related to the development of a series of new, as well as already existing, experimental methods. A good example in this context is microscopy, where the advancements on atomic force^1^ and electron microscopes^2^ allowed access to previously unknown territories. Another good example is the area of photoelectron spectroscopy^3^, which has been one of the biggest breakthroughs for surface science. With the development of synchrotron X-ray sources providing flexible wavelength and high brilliance, previously unachieved sensitivity was reached for compositional and structural studies of surfaces and interfaces.

Using surface scattering methods, the electron density across interfaces can be extracted with Å resolution from specular (incident beam angle equals exiting beam angle) X-ray reflectivity. Further, off-specular or gracing incidence scattering provides information about in-plane ordering and fluctuations. In particular the high brilliance of synchrotron X-ray sources allows grazing incidence small and wide-angle X-ray scattering (GISAXS and GIWAXS) in a routine way^4,5^. There are now about 30 synchrotron beamlines offering GISAXS as a technique as well as an increasing number of laboratory sources providing a GISAXS mode.

Compared to electrons and photons, neutrons are sensitive to light elements including hydrogen, can penetrate deep into matter and provide a direct measure of the magnetic induction in materials. Moreover, the sensitivity to the nucleus enables contrast variation experiments by isotope substitution (e. g. hydrogen versus deuterium), which is a key in soft matter research. The drawback of neutron scattering methods, however, is that they are only available at centralized facilities and that the brilliance of neutron sources is limited. This is particularly relevant for small sample volumes or interface and surface scattering studies.

Specular neutron reflectometry (NR) is a well-established technique that provides information about the density profile of isotopes across an interface. For more details on the method and a recent review see ref. ^6^. As for X-rays, lateral fluctuations can be investigated via, so called, off-specular and grazing incidence small angle neutron scattering (GISANS)^7,8^. The GISANS technique is in between two well-established methods, NR and small angle neutron scattering (SANS). Today, about 30 neutron reflectometers are operating around the world, comparable to the number of SANS beamlines and many GISANS experiments are performed on not optimised instruments. Challenges for high data quality are a neutron beam collimation matching to the sample in-plane and out-of-plane structure as well as optimized beam shapes and detector geometries. The first paper on GISANS was only published in the mid 90’s^9^. It took another 10 years until a dedicated instrument, Ref-SANS at the MLZ (Garching, Germany)^10^ became available. However, this instrument faces several design compromises, such as a twisted guide and focusing optics, which are not separated from the multi-chopper system. To combine NR with good in-plane resolution several beams are sent onto the sample surface and focused onto the detector. As a result, large sample areas are required and the instrument performs more like a reflectometer. An attempt to make effective use of divergent neutron beams is the use of spin polarised neutrons together with magnetic fields for Lamor precession to encode the scattering angle with so-called spin-echo-resolved grazing incidence scattering (SERGIS)^11–13^. Instead of spatial angular resolution this method measures the depolarization of the beam to record very week scattering of neutrons in the sample plane. This allows a relaxed collimation of the beam and a significant increase in neutrons impinging on the sample. However, even though this method is routinely available at Offspec at the ISIS Neutron and Muon source (Oxfordshire, UK)^14^, it is not widely used as it is technically demanding and requires advanced data processing.

In the present study we perform GISANS experiments by a simple change of the sample geometry from vertical (used for NR) to horizontal at the neutron reflectometer Super ADAM (Institute Laue-Langevin, Grenoble, France). In this configuration, the incident neutron flux becomes enhanced but we avoid the challenges of Ref-SANS or the SERGIS method. We use the full divergence delivered by the monochromator along the surface normal but high Q resolution in the plane of the interface. Using this configuration, we show that high quality, one-dimensional, GISANS data can be collected approximately 100 times faster compared to the ordinary vertical scattering geometry. We measure particle shapes as well as the correlations between buried iron inclusions in aluminium oxide films, self-assembled silica spheres on a silicon substrate and, what is more unique, on a free water surface.

## Experimental details

**Figure 1:**
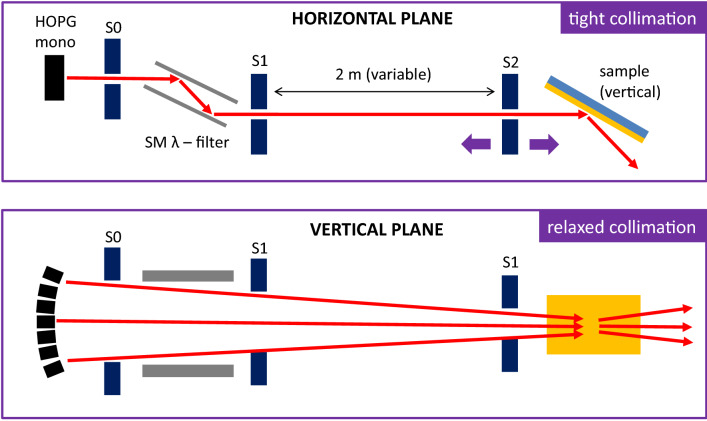
Sketch of the focusing and collimation systems of the instrument Super ADAM.

Super ADAM is a neutron reflectometer using a monochromatic beam. The neutrons of wavelength 5.21 Å are selected from the guide by a HOPG monochromator allowing an excellent wavelength resolution of Δλ/λ = 0.4%. More details on the instrument can be found elsewhere^15,16^. For reflectometry measurements, the neutron beam is reflected of a vertically mounted sample surface by scanning the sample and scattering angle. In this case and to take maximum benefit of the neutrons delivered by the H53 guide, the incident neutron beam is focused vertically, using the full height of the neutron monochromator of 150 mm and consisting of seven individually aligned HOPG crystals (see Fig. 1, lower panel). The neutron reflectivity measurement is done along the horizontal direction, where the Q resolution may be very high (see Fig. 1, upper panel).

For GISANS, on the other hand, the incident beam needs to be collimated along the sample in-plane direction (vertical direction), which results in a significant loss of incident neutron flux. In the past, this mode was used to study the self-assembly of polymer micelles at solid substrates on Super ADAM^17,18^.

**Figure 2:**
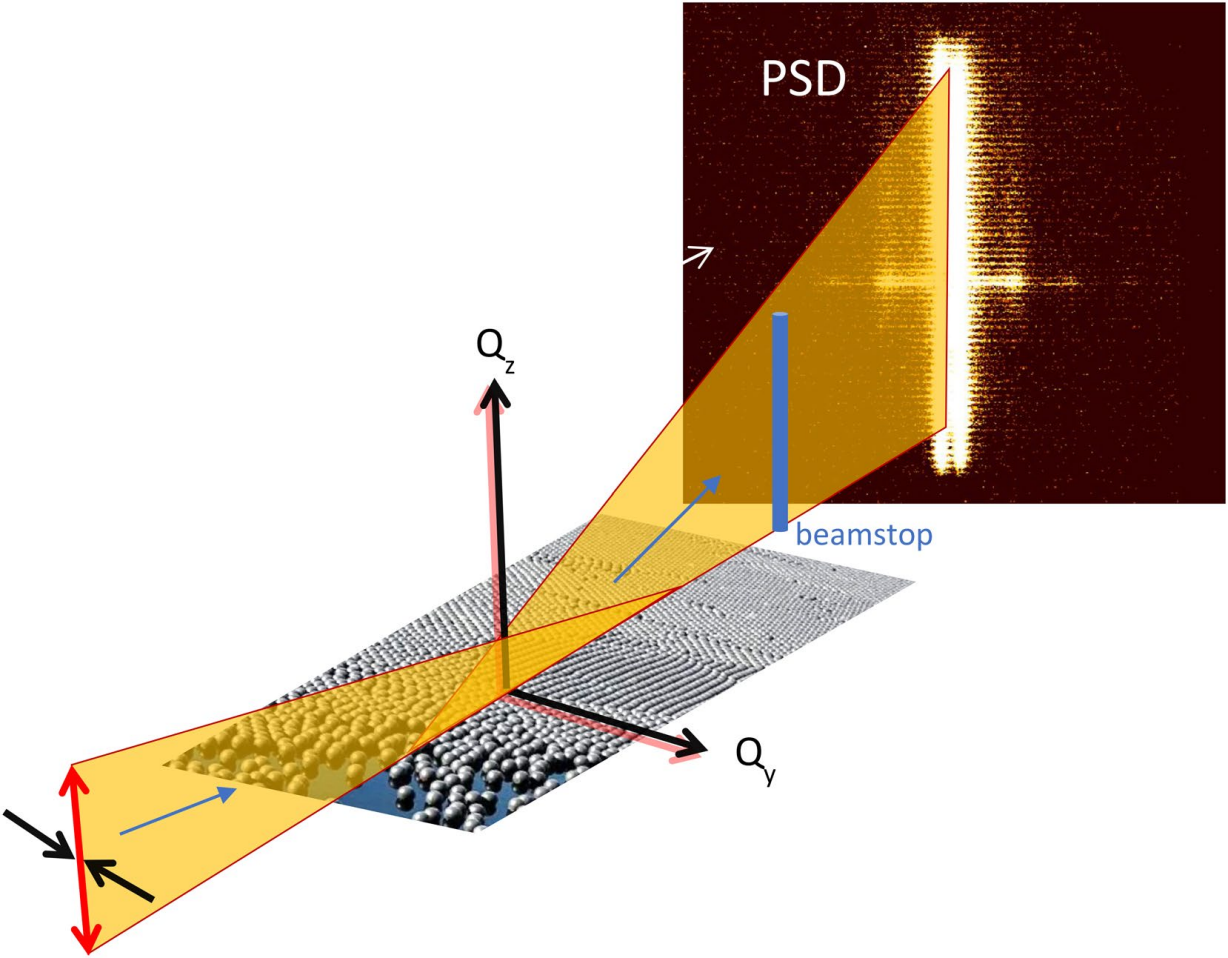
Sketch of the scattering geometry for one-dimensional (π-GISANS) studies.

An alternative way to do GISANS measurements is to turn the sample by 90 degrees and scatter from a horizontal sample surface (Fig. 2). In this case, one takes benefit of the inherently good resolution along the horizontal direction while using the full divergence from the monochromator along the vertical direction. This results in a range of incident beam angles, covering about two degrees, adjustable by the distance and vertical opening of the collimating slits. Note, effectively the accepted divergence in this geometry is defined by the height of the monochromator and the sample footprint. The potential gain of our method can be estimated from the source size, in our case the usable part of the monochromator. For GISANS done in the traditional way a source size of 1.5 mm may be used whereas we use 15 cm, resulting in a potential gain in flux of two orders of magnitude. However, the large incident divergence results in a loss of depth sensitivity^19^. Still the method has all the advantages of neutron scattering experiments benefiting from isotope contrast variation, sensitivity to light elements and the capability to probe buried interfaces as well as the magnetic induction. This is not the case for microscopy-based techniques such as atomic force microscopy and scanning electron microscopy. Moreover, and complementary to microscopy methods, our approach allows to extract information averaged over large sample sizes. Already today, many GISANS experiments are done on SANS instruments using relatively large incident beam angles and looking on the evanescent wave in the outgoing beam, or so-called horizontal line cuts^20^. This is very similar to our method but without the additional benefit of flux enhancement due to the anisotropic divergence of the incident beam. For single interfaces or thin films, the limited depth resolution is not a drawback as the only part scattering laterally in the sample is the film of interest. Moreover, depth resolution can be recovered with additional neutron reflectivity measurements with the advantage that both measurements together can still be performed with reasonable data acquisition times.

Figure 2 depicts a sketch of the scattering geometry. The incident beam is divergent along the surface normal, z-direction. Along the horizontal direction the collimating slits 1 and 2 usually used for reflectometry measurements define the divergence and were set to 1 mm with the distance between them being 2010 mm to provide high Q resolution of ΔQ = 2.4 * 10^–3^ Å^−1^ (FWHM). Note, as the wavelength resolution on Super ADAM is very high the resolution at small scattering angles is defined by the beam divergence. However, on Super ADAM the beam incident to slit 1 is precollimated by an additional slit and a double bounce wavelength filter. This results in an experimentally determined Q resolution of ΔQ = 5(1) * 10^–4^ Å^−1^ (FWHM) for the geometry used in our experiments and extracted from fits to neutron reflectivity data. After reflection from the horizontal surface the GISANS signal is registered by a position sensitive detector, manufactured by Denex and of size 300 mm × 300 mm with resolution 2.5 mm at a distance of 3000 mm from the sample. The direct and specular reflected beams are absorbed using a beamstop. Figure 2 shows a sketch of a detector image, taken with spherical particles spread and densely packed on a D_2_O surface. The horizon/Yoneda region^21^, where the incident and exiting wave fields are in phase resulting in enhanced intensity, is clearly visible as brighter horizontal stripe. In addition, side peaks, along Q_y_, and resulting from the structure factor of the particles are well resolved and visible as well. The scattering pattern is smeared out along the z-direction as a large incident beam divergence is used and for further analysis the detector image may be integrated along the vertical direction, resulting in cuts focusing on the in-plane scattering. The vertical integration averages out potential variations in intensity resulting from the structure of the monochromator or detector. As the depth resolution of this method is very limited, the approach might be seen as one-dimensional GISANS, that we call pseudo-GISANS or π-GISANS. In this manuscript, we show raw data. Incident beam normalization is not required on a monochromatic instrument for a single point measurement as long as no absolute cross sections are evaluated. Detector corrections, noise or flat field, might further improve the data quality but was omitted to underline the simplicity and potential of the method.

## Results

**Figure 3:**
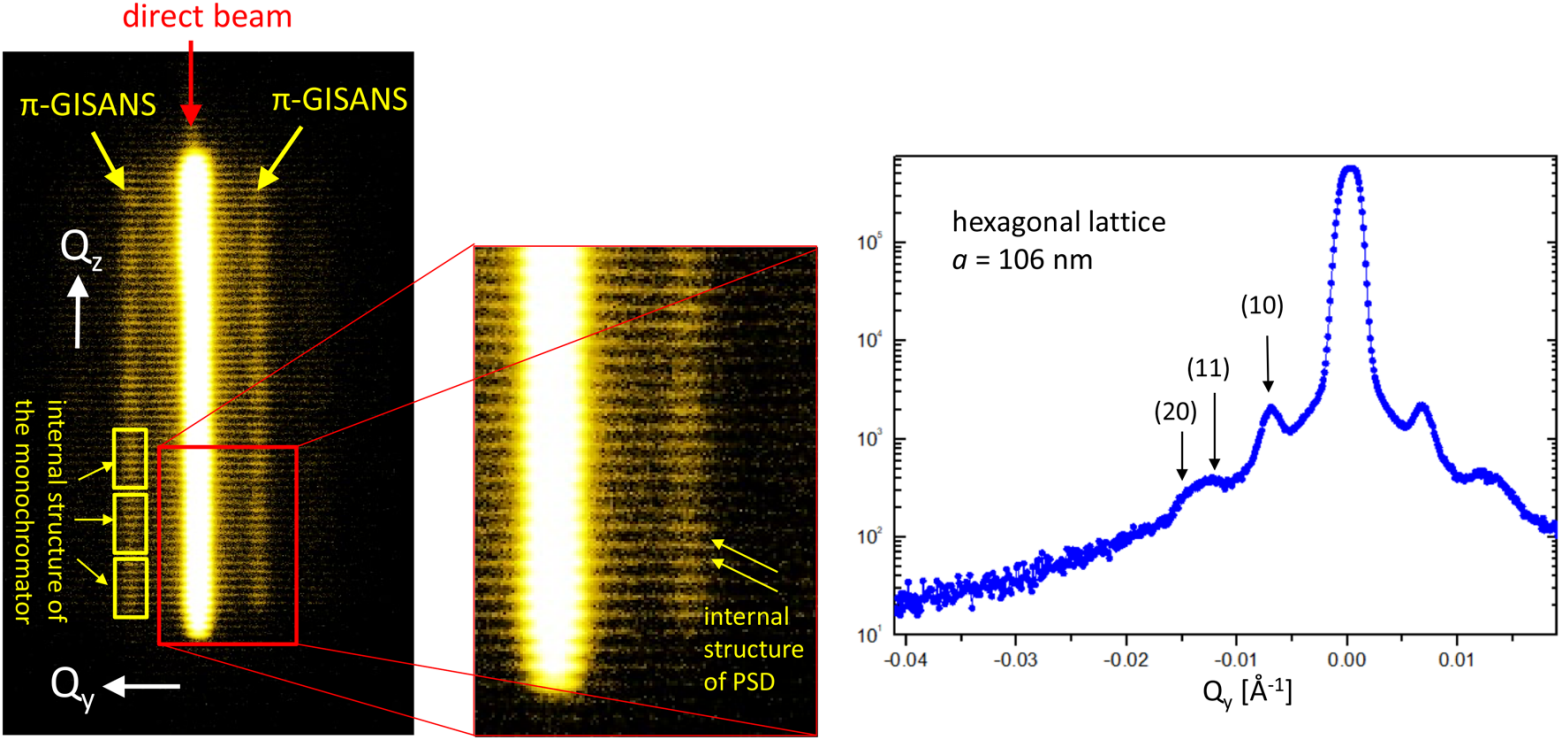
π-GISANS data taken on Super ADAM with a sample of a porous aluminum oxide film with the pores filled with iron. The left panel depicts the image on the PSD of Super ADAM with the individual monochromator crystals well visible. The center panel depicts a zoom into the detector image to highlight the internal structure of the wire detector. The panel on the right hand side depicts the vertically integrated data revealing Bragg scattering from the iron arranged in a hexagonal lattice.

As a first sample we measured a solid film of porous aluminum oxide with pores filled by iron, providing excellent contrast for neutrons. The sample was capped with a germanium capping film to prevent oxidation therefore making studies using microscopy challenging. On this sample, a good quality π-GISANS signal was obtained in less than 30 min. Figure 3, left panel, depicts the raw detector image. The bright vertical line in the center is the fraction of the direct beam passing the beamstop. The GISANS signal is visible to the left and right of the direct beam. As the sample together with the collimating slit 2 (vertical opening 2 mm) acts as a line shaped pinhole camera, the seven individual monochromator crystals are imaged on the detector, demonstrating the focusing performance of the instrument as well as the identical size and quality of the HOPG crystals. The central panel of Fig. 3 depicts a zoom into the detector image to highlight the fine structure of the detector itself. The narrow horizontal lines relate to the wire structure of the detector used as delay lines to resolve the position where the neutron is absorbed by the He gas inside the detector. Note, as the signal is integrated along the vertical direction for the analysis of the π-GISANS data, the vertical variations in intensity resulting from the instrument settings are averaged out. However, this data can be used to investigate the performance of the instrument and verify the focusing of the monochromator.

The right panel in Fig. 3 depicts the vertically integrated π-GISANS intensity plotted versus in-plane momentum transfer, Q_y_. At least three Bragg peaks can be identified and indexed by a hexagonal 2D lattice with a lattice parameter of 106 nm, which is in excellent agreement with the 100 nm aimed for during sample production. Note, the (11) and (20) reflections for a hexagonal lattice are very close to each other, difference in Q less than 15%, and therefore hard to separate.

As a second example, we investigated silica spheres of diameter 50 nm, 100 nm and 200 nm deposited by a modified Langmuir-Schaefer method on a silicon substrate^22^. Spherical, non-porous silica nanoparticles dispersed at 5 mg/mL in an ethanolic solution containing 1 mM CTAB were sonicated and spread onto a clean water surface. After adjusting the surface pressure to ensure close particle packing while avoiding collapse of the monolayer, the monolayer was lowered onto a previously submerged silicon crystal (60 × 80 mm) by slowly removing water from the trough until the air/water interface crossed the solid interface.

**Figure 4:**
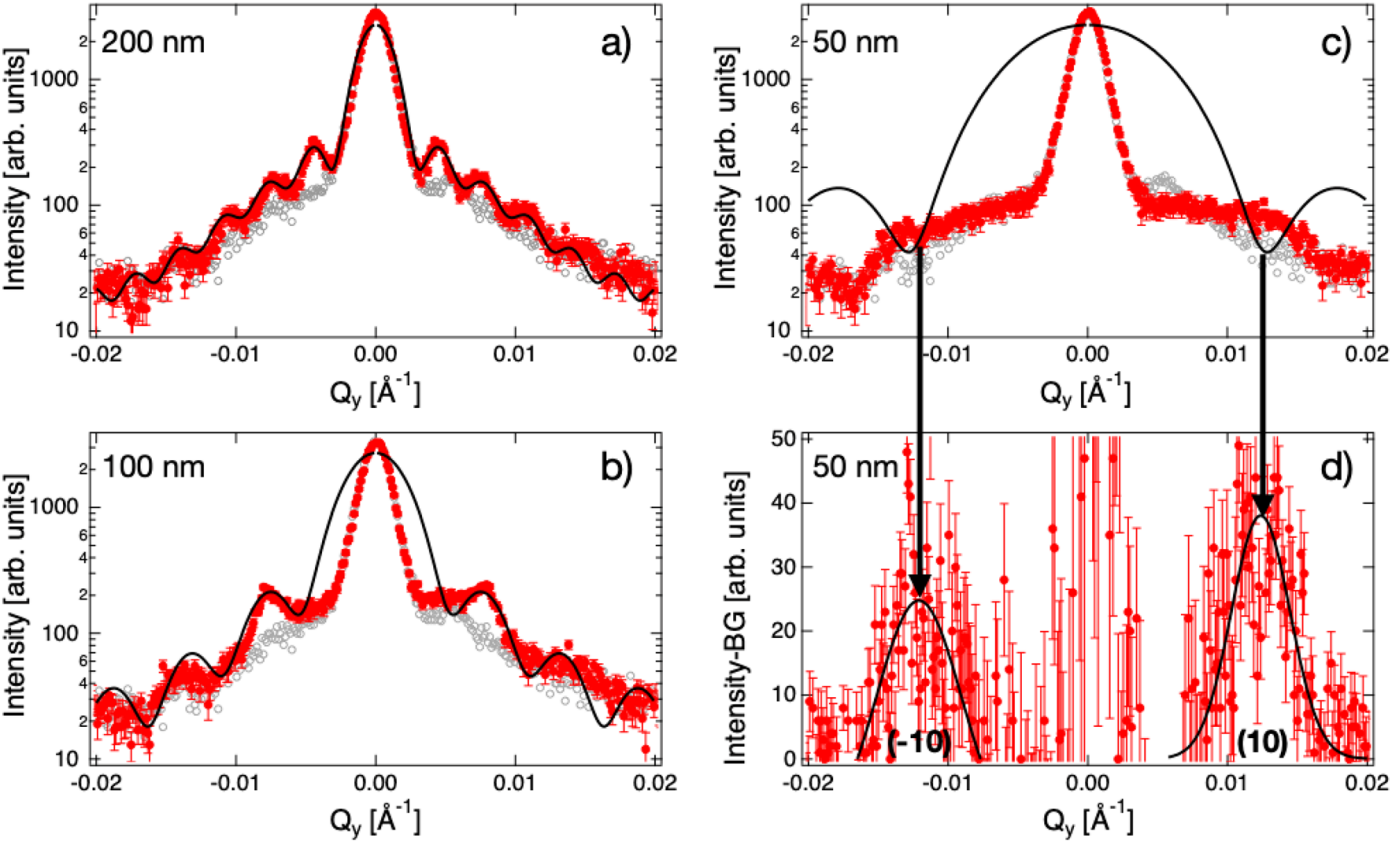
π-GISANS data taken on Super ADAM with silica particles, of size 200 nm (**a**), 100 nm (**b**) and 50 nm (**c**, **d**), deposited on a silicon wafer and shown as red circles. The grey open circles symbolise a measurement performed with a bare silicon substrate. The solid black lines represent simulations of the form (**a**, **b**, **c**) and structure factor (**d**) of the particles.

**Figure 5:**
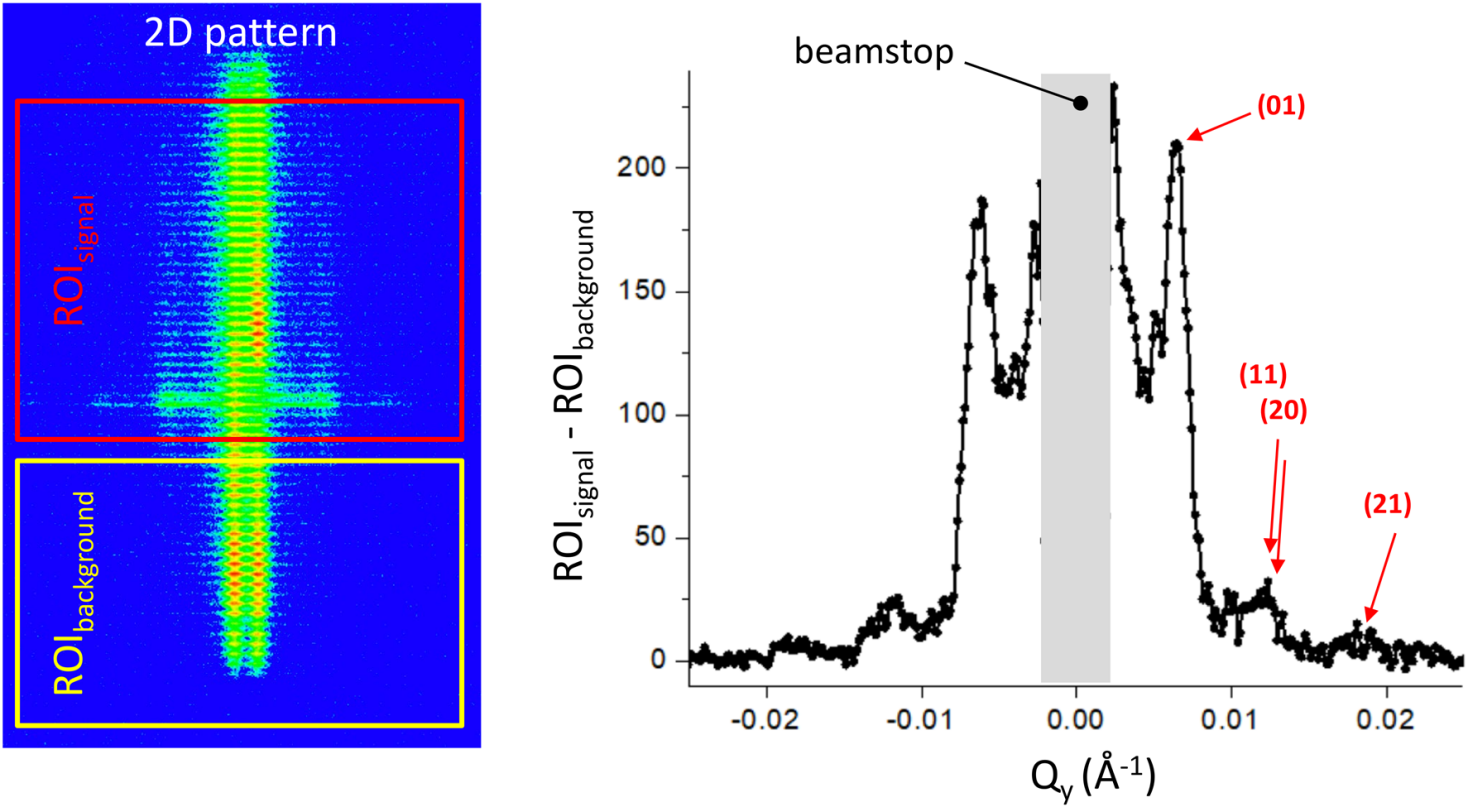
π-GISANS data taken on Super ADAM for 100 nm silica particles self-organised on a D_2_O surface. The panel to the left depicts the raw 2D-detector image with the regions of interest for the signal and background indicated. The panel to the right is the same data but vertically integrated over the respective regions of interest. The GISANS signal minus the background is plotted versus Q_y_ on a linear scale.

The logarithm of the neutron intensity collected on the PSD detector with the resulting Silica nano-particle samples is plotted versus the in-plane momentum transfer Q_y_ in Fig. 4a-c. The data is integrated vertically in the region of interest (above the sample horizon) as shown in Fig. 5. A bare silicon wafer was measured at the same instrument settings as background measurement and is shown by the grey dots. The counting time for each of the π-GISANS patterns was 5 h. For the data taken with the 200 nm particles (Fig. 4, panel a), clearly several maxima are visible in the π-GISANS signal. It turns out that the position of the first minima is almost exactly at a Q_y_ value of 2π/200 nm as expected for a particle form factor but not for a structure factor of a densely packed layer. Moreover, a (11) reflection as expected for hexagonal packing is absent. Therefore, we simulated a particle form factor for 200 nm hard spheres according to the following equation to describe the data:

1 $$I = A\left( {\frac{\sin (RQy)}{{RQy}}} \right)^{2} + Be^{{ - \frac{{Q_{y}^{2} }}{{w^{2} }}}} + C$$

The first term in Eq. (1) is the form factor of hard sphere particles of radius R, the second term assumes a Gaussian background from, e.g. tails of the direct beam or blooming of the detector, due to the high count rate close to the direct beam and the last term, C, assumes a constant background from dark noise. The result of the calculation is shown in the panel as black line and is in excellent agreement with the data assuming a particle diameter of 2R = 200 nm. In our simulations, we did not include the Q resolution of the instrument. Including the Q resolution via a convolution would change the width of the oscillations and the depth of the minima between them but would not affect their positions. The good fit to the central peak at Q = 0 is by coincidence, as this part of the detector is shaded by the beamstop, which otherwise would be hit by the reflected/direct beam.

The same simulation was done for the 100 nm particles (Fig. 4, panel b). This time the simulation describes the data less well. All oscillations are smeared out and we have to assume a particle diameter of 116 nm, rather than the expected 100 nm, to get a reasonable agreement with the data. The discrepancy between data and simulations may be explained by the background or by contributions from the structure factor broadening the peaks and shifting them to lower Q_y_ values. Note, the structure factor scales with the number of coherently scattering particles squared, while the from factor goes linear with the total number of scatterers.

The effect of the structure factor is very clearly visible for the 50 nm particles (Fig. 4, panel c). In this case there is an evident mismatch between the data and calculation of the form factor of 50 nm spheres where maxima in the data are at the Q_y_ positions where the simulation predicts minima. To highlight this effect Fig. 4, panel d, depicts the data taken with the 50 nm particles with the background from the bare silicon (grey dots) subtracted and plotted on a linear scale to highlight the structure factor scattering. In this representation clearly two peaks are visible. To determine their position in Q_y_ we fitted two Gaussians and extract a nearest neighbor distance of 51.4 nm, which is close to the particle diameter. The slightly larger value may relate to disorder or the coating of the particles. Overall, we find a trend of better ordering for smaller particles. Note, it is not possible to evaluate the intensities of the peak around Q = 0 as the beamstop shadows this region of the detector, which would otherwise be hit by the reflected, direct and not scattered, beams.

To get a first handle on the self-assembly process of the particles during the Langmuir-Schaefer process we have spread the identical silica particles of size 100 nm on a water (D_2_O) surface. The measurements were done using a simple water basin consisting of a silica frame glued on a silicon block and mounted on an active anti-vibration device from Halcyonics, avoiding any surface waves on the liquid surface. The amount of nanoparticles to deposit was calculated such that the total projected area of the spheres arranged in a monolayer is equal to that of the water surface available in the trough, to from a densely packed layer. Particles were dispersed at 5 mg/mL in ethanol in the presence of 1 mM CTAB and added dropwise to the D_2_O surface. As background measurement, we used the intensity transmitted through the sample and subtracted it from the GISANS signal reflected from the surface. For the respective regions of interest on the detector we refer to Fig. 5 (left panel). This method allows plotting of the π-GISANS signal on a linear scale to highlight the structure factor peaks. Note, the attenuation of the beam in D_2_O and silica is small and we did not use a normalization factor. The result is depicted in Fig. 5 (right panel) with the intensity plotted on a linear scale against the in-plane momentum transfer Q_y_. Several Bragg reflections are resolved and can be indexed by assuming a hexagonal dense packed monolayer of 100 nm silica particles. As the scattering from the structure factor is more intense than the one from the form factor, data of acceptable quality could be obtained in 2 h.

The detector images reproduced in Figs. 2, 3 and 5 reveal a peculiar difference. For the iron inclusions the detector image (Fig. 3) is homogenous along the vertical direction, while for the silica particles (Fig. 2 and 5) the sample horizon is clearly visible as horizontal stripe. This is related to the fact that the iron inclusions are buried in the substrate while the silica particles are deposited on the substrate. For the first case, neutrons may be totally externally reflected above the actual sample with only the evanescent wave field penetrating. For the second case the neutrons are reflected below the silica spheres at the silicon surface resulting in more pronounced resonant enhancement or so-called Yoneda scattering in the outgoing beam. Note, the reflected beam is masked in both cases and therefore invisible. Recently we have done a NR study of silica spheres sandwiched between silicon and D_2_O. In this case the silica particles form a resonator layer and the GISANS signal becomes even more enhanced as at the resonant conditions one is extremely sensitive to any scattering^23,24^. This demonstrates that even with the π-GISANS method some depth information is accessible and that for a full quantitative analysis of GISANS data wave field calculations in the distorted wave Born approximation are required^25^ but appropriate length scales can be extracted from the peak positions only.

## Summary

We show that one-dimensional π-GISANS measurements can be done using a divergent incident neutron beam and integrating the detector images along the out-of-plane direction. In this way we get a significant increase in incident-beam intensity but compromise on depth resolution. For single films and interfaces this is acceptable. Note, we still take benefit of the advantages of neutrons, which are sensitive to isotopic contrast and the magnetic induction. The method also allows straight forward investigations of buried interfaces which cannot be achieved with any microscopy based method. We show that this method works well on the monochromatic, high resolution, neutron reflectometer Super ADAM and resolve the lateral structure of iron inclusions in an aluminum oxide matrix. In addition, we resolve the particle form factor of silica spheres as well as the structure factor from their hexagonal arrangement on a silicon as well as water substrate with reasonable counting times. The intensity gain on Super ADAM for our method is approximately two orders of magnitude compared to the conventional vertical geometry. Note, Super ADAM has very high wavelength resolution of Δλ/λ = 0.4% but our method is equally applicable on instruments with relaxed wavelength resolution. Actually, the high wavelength resolution limits the incident beam flux and we expect even larger gains if the method is implemented on instruments with relaxed Δλ/λ and wider beams, as used on most SANS instruments. In this case additional optics may be used to focus the beam in the out of-plane direction to provide a line focus. Like this the full height of the beam on a SANS instrument may hit the sample surface avoiding over-illumination, which usually results in a considerable loss of incident beam intensity. We expect that with an optimised focusing device, improved background shielding and optimized sample environment counting times will be significantly reduced and gains in intensity similar to the one on Super ADAM can be expected. In this way counting times should come into the minute range, rather than hours, which are required today. This will unlock new science by running contrast variation experiments or wider ranges of experimental parameters, as for example temperatures or magnetic fields/hysteresis loops. Potentially our method may even enable kinetic studies, which are not possible today. Our method may be applied to any scientific field already addressed by grazing incident neutron scattering methods, such as quantum effects, drug delivery, biomembranes, solar and fuel cell research, to name just a few and has the potential to make GISANS a standard tool in interface science.

## References

[1] Giessibl FJ (2003). Advances in atomic force microscopy. Rev. Mod. Phys..

[2] Egerton RF (2016). Physical Principles of Electron Microscopy.

[3] Hüfner S (2003). Photoelectron Spectroscopy.

[4] Levine JR, Cohen JB, Chung YW, Georgopoulos P (1989). J. Appl. Cryst..

[5] Hexemer A, Bras W, Glossinger J, Schaible E, Gann E, Kirian R, MacDowell A, Church M, Rude B, Padmore H (2010). J. Phys. Conf. Ser..

[6] Wolff M, Gutfreund P, Aliofkhazraei M, Nasar A, Chipara M, Laidani N, De Hosson JM (2020). Neutron reflectivity for the investigation of coatings and functional layers. Handbook of Modern Coating Technologies, Characterisation Techniques.

[7] Wolff M., Grazing incidence scattering, EPJ Web of Conferences **188**, 04002 (2018).

[8] Jaksch S, Gutberlet T, Müller-Buschbaum P (2019). Grazing-incidence scattering—status and perspectives in soft matter and biophysics. Curr. Opin. Colloid Interface Sci..

[9] Hamilton WA, Butler PD, Baker SM, Smith GS, Hayter JB, Magid LJ, Pynn R (1994). Shear induced hexagonal ordering observed in an ionic viscoelastic fluid in flow past a surface. Phys. Rev. Lett..

[10] Kampmann R, Haese-Seiller M, Marmotti M, Burmester J, Deriglazov V, Syromiatnikov V, Okorokov A, Frisius F, Tristl M, Sackmann E (2002). The novel reflectometer REFSANS for analyses of liquid and soft surfaces at the new research reactor FRM-II in Munich, Germany. Appl. Phys. A.

[11] Felcher SGP, te Velthuis SGE, Major J, Dosch H, Anderson Ch, Habicht K, Keller Th (2002). Spin-echo resolved grazing incidence scattering (SERGIS) of cold neutrons. SPIE.

[12] Pynn R, Fitzsimmons MR, Rekveldt MTh (2002). Optimization of neutron scattering instrumentation using neutron spin echo: Application to the discrimination of diffuse scattering in neutron reflectivity experiments. Rev. Sci. Instrum..

[13] Vorobiev A, Major J, Dosch H, Müller-Buschbaum P, Falus P, Felcher GP, te Velthuis SGE (2011). Phase and microphase separation of polymer thin films dewetted from silicon—A spin-echo resolved grazing incidence neutron scattering study. J. Phys. Chem. B.

[14] Dalgliesh RM, Langridge S, Plomp J, de Haan VO, van Well AA (2011). Offspec, the ISIS spin-echo reflectometer. Phys. B Cond. Mat..

[15] Vorobiev A, Devishvili A, Palsson G, Rundlof H, Johansson N, Olsson A, Dennsion A, Wolff M, Giroud B, Aguettaz O, Hjörvarsson B (2015). Recent upgrade of the polarized neutron reflectometer Super ADAM. Neutron News.

[16] https://www.ill.eu/users/instruments/instruments-list/superadam/characteristics.

[17] Wolff M, Scholz U, Hock R, Magerl A, Zabel H (2004). Crystallisation of micelles at chemically terminated surfaces. Phys. Rev. Lett..

[18] Wolff N, Gerth S, Gutfreund P, Wolff M (2014). Temperature dependent cubic and hexagonal packing in micellar structures. Soft Matter.

[19] Adlmann F, Herbel J, Korolkovas A, Bliersbach A, Toperverg BP, van Herck W, Palsson GK, Kitchen B, Wolff M (2018). Depth resolved grazing incidence neutron scattering experiments from semi-infinite interfaces: A statistical analysis of the scattering contributions. J. Phys. Cond. Mat..

[20] Müller-Buschbaum P (2013). Gracing incidence small angle neutron scattering: Challenges and possibilities. Polym. J..

[21] Yoneda Y (1963). Anomalous surface reflection of X-rays. Phys. Rev..

[22] Chitu L, Siffalovic P, Majkova E, Jergel M, Vegso K, Luby S, Capek I, Satka A, Perlich J, Timmann A, Roth S (2010). Modified Langmuir–Blodgett deposition of nanoparticles-measurement of 2D to 3D ordered arrays. Meas. Sci. Rev..

[23] Perrichon A, Devishvili A, Komander K, Palsson GK, Vorobiev A, Laven R, Karlsson M, Wolff M (2018). Resonant enhancement of grazing incidence neutron scattering for the characterization of thin films. Phys. Rev. B.

[24] Wolff M, Devishvili A, Dura JA, Adlmann FA, Kitchen B, Palsson GK, Palonen H, Maranville BB, Majkrzak ChF, Toperverg BP (2019). Nuclear spin incoherent neutron scattering from quantum well resonators. Phys. Rev. Lett..

[25] Hafner A, Gutfreund P, Toperverg BP, Jones AOF, de Silva JP, Wildes A, Fischer HE, Geoghegan M, Sferrazza M (2021). Combined specular and off-specular reflectometry: Elucidating the complex structure of soft buried interfaces. J. Appl. Cryst..

